# Bioactive Antioxidants from Avocado By-Products: Mechanistic Study and Laboratory-Scale Extraction Optimization

**DOI:** 10.3390/antiox14101225

**Published:** 2025-10-11

**Authors:** Ziyao Xin, Yicheng Gao, Leiyu He, Zhilong Xiu, Lihui Sun

**Affiliations:** 1 School of Chemical Engineering Ocean and Life Sciences, Dalian University of Technology, Panjin 124221, China; 2 School of Bioengineering, Dalian University of Technology, Dalian 116000, China

**Keywords:** avocado by-products, antioxidants, ultrasound-assisted extraction, molecular docking, central composite design

## Abstract

This study aimed to develop an environmentally friendly and relatively efficient method for extracting natural antioxidants from avocado by-products while investigating the antioxidant mechanisms of their core bioactive components on multiple dimensions. In vitro antioxidant assays (ABTS, FRAP, SAFR, SFR, ORAC, DPPH) demonstrated that flavonoid procyanidin was the primary antioxidant component in avocado seeds, exhibiting the strongest activity (DPPH EC_50_ = 3.6 µg/mL). The Hill model indicated a positive synergistic effect (*n* = 3.1). Chemical and molecular mechanism analyses revealed that avocado seeds exert antioxidant activity predominantly through hydrogen atom transfer (HAT) and electron transfer (ET) pathways. The model predictions suggested procyanidins may stably bind to protein targets in the Keap1-Nrf2 pathway and NOX2 via hydrogen bonding, hydrophobic interactions, and π-cation interactions. Furthermore, response surface methodology (RSM) was employed to optimize the extraction process of avocado seed antioxidants in an ethanol-water system. This study underscores the considerable health benefits and antioxidant capacity of avocado by-products, supporting their promising application in functional foods formulations.

## 1. Introduction

Avocado (*Persea americana Mill.*), primarily cultivated in tropical and subtropical regions, has a global annual production exceeding three million tons [1]. Renowned for its creamy flesh and rich content of monounsaturated fatty acids, current research predominantly focuses on the flesh, whereas studies on avocado by-products remain limited. Evidence suggests that these by-products contribute approximately 70% of the fruit’s total antioxidant capacity and account for nearly 30% of its weight [2]. Despite their potential value, avocado processing by-products are frequently discarded. According to Nyakang et al., approximately 2.42 million tons of avocado seeds are discarded annually as production waste, resulting in significant resource waste and environmental pollution [3]. In recent years, reducing industrial waste and promoting sustainable resource management have become key priorities for relevant authorities.

Avocado by-products serve as a natural, low-cost source of bioactive compounds. They contain significant amounts of dietary fiber, monounsaturated fatty acids, and resistant starch, as well as a wide range of polyphenolic and polymeric compounds, including anthocyanins, hesperidin, quercetin, catechins, condensed tannins, flavonoids, and terpenoids [4]. Research indicates these compounds exhibit potent biological activities, including anticancer, anti-inflammatory, antioxidant, antidiabetic, and cholesterol-lowering effects. The anti-inflammatory activity of polyphenols in vivo is closely linked to their potent antioxidant capacity, potentially regulating cellular responses and oxidative stress by scavenging free radicals [5]. Moreover, dietary intake of foods with high antioxidant activity helps mitigate the adverse effects of free radicals on the human body and reduces the incidence of health issues associated with oxidative stress [6]. Therefore, given that plant extracts are favored by consumers for their dual properties of bioactivity and distinctive flavor, their application in the development of functional foods has emerged as a new industry trend [7].

Currently, industrial extraction methods for bioactive compounds predominantly rely on conventional organic solvent reflux techniques. However, the use of conventional solvents such as diethyl ether, acetone, and chloroform not only limits their applicability in the food industry but also gives rise to multiple concerns, including health hazards, environmental toxicity, and resource depletion [8]. Therefore, there is an urgent need to advance research on novel bioextraction technologies and alternative bio-based solvents in the field of extraction. Recent studies have demonstrated that hydrodynamic cavitation (HC) is a promising green and sustainable technique for bioextraction, with broad applicability in plant resource utilization and industrial-scale processes [9]. This study aims to evaluate the feasibility of replacing conventional solvents with safe and highly biocompatible alternatives—particularly biodegradable and readily available solvents—at the laboratory scale. Ethanol, as a low-toxicity, renewable, and versatile solvent, demonstrates significant advantages for the extraction of compounds with complex or poorly characterized compositions, demonstrates high efficiency in polyphenol extraction. This efficiency stems from ethanol’s ability to degrade polysaccharides in plant cell walls, thereby promoting the release of phenolic compounds [10]. Currently, ethanol is one of the most widely used solvents in the extraction processes of the food industry. However, research on the integration of ultrasound-assisted extraction (UAE) with safe and renewable solvents for the recovery of bioactive compounds from avocado by-products remain limited.

Existing research on the bioactive compounds in avocado by-products and their antioxidant properties remains limited. The development and utilization of these by-products are still in their infancy. Therefore, in-depth analysis of their nutritional and bioactive components is of significant importance. This study aims to evaluate the potential utilization value of avocado by-products, elucidate the chemical basis and possible molecular mechanisms underlying their antioxidant activity, and determine the optimal green extraction protocol by combining ultrasonication and thermal assistance using the RSM method. This work aims to provide a reference for the subsequent optimization of related extraction processes and for researchers in the food industry engaged in the development of multifunctional foods.

## 2. Materials and Methods

### 2.1. Chemicals

All chemical reagents and standards used in this experiment were of analytical grade and chromatographic grade. Trolox (≥98%) was purchased from Aladdin Holdings Group Co., Ltd. (Shanghai, China). Catechin (≥97%), quercetin (≥98%), rutin (≥95%), ammonium ferric sulfate dodecahydrate (≥99.9%), proanthocyanidin (≥98%), amygdalin (≥99%); 2,2′-Biphenyl-1-pyridinehydrazine (≥96%), 3wt% hydrogen peroxide, trimethylamine (≥99%), sodium fluorescein (≥85%), 2,2-azobis(2-methylpropylimidazole) dihydrochloride (≥99%), tetrazolium red (≥95%), formic acid (HPLC grade), methanol (HPLC grade), Folin–Ciocalteu phenol reagent, gallic acid (>99%), ethanol were purchased from Shanghai Macklin Biochemical Co., Ltd. (Shanghai, China). Vanillin (>99%), Salicylic acid, L-Ascorbic acid and ABTS (≥99%) were purchased from Sangon.

### 2.2. Materials

The experimental material used in this study was Hass avocado, sourced from Baoshan City, Yunnan Province, China. After procurement, the raw material was stored at 4 °C for subsequent testing. A portion of the avocado seeds underwent processing: dried samples were ground in a mortar until reduced to a fine, particle-free powder. This powder was stored at −20 °C for later analysis.

### 2.3. Nutritional Analysis

The nutritional components of avocado were determined according to the AOAC (2000) method, including moisture (method 925.10), ash (method 942.05), protein (method 923.03; N × 6.25), crude fat (method 920.39), total acidity (method 942.15), and fiber content (method 969.09). Total sugar content was determined using the phenol-sulfuric acid method as referenced by Yue et al. [11].

### 2.4. Analysis of Bioactive Components

#### 2.4.1. Total Polyphenol Content

The total polyphenol content of the samples was determined using the Folin–Ciocalteu method, adapted from Rumpf et al. with minor modifications [12,13]. The extract reacted with Folin–Ciocalteu reagent and Na_2_CO_3_ in the dark for 2 h. Absorbance was measured at 765 nm using a microplate reader (Spectra Max M2e, Molecular Devices, San Jose, CA, USA). A standard curve was plotted using gallic acid standards ranging from 0 to 200 mg/L (R^2^ = 0.995). Total phenolic content results were expressed as milligrams of gallic acid equivalent per gram (mg GAE/g).

#### 2.4.2. Total Flavonoid Content

The total flavonoid content in the samples was determined using the NaNO_2_-Al(NO_3_)_3_ method, adapted from Siti Atikah et al. [14,15]. The extract was sequentially reacted with NaNO_2_, Al(NO_3_)_3_, and NaOH solutions. Using rutin standards at concentrations ranging from 0 to 1 mg/mL (R^2^ = 0.997) as a reference, with measurements at 510 nm. Total flavonoid content was expressed as rutin equivalents per milligram (mg RE/g).

#### 2.4.3. Crude Polysaccharide Content

Crude polysaccharides in avocado were detected using the phenol-sulfuric acid method, following the protocol of Wang et al. [16]. Samples were extracted via water extraction and ethanol precipitation, the extract was thoroughly mixed with phenol-sulfuric acid reagent, reacted in a boiling water bath for 20 min, cooled on ice, and then measured for absorbance at 490 nm. Using anhydrous glucose standards in the concentration range of 0–100 mg/L (R^2^ = 0.999) as a reference. Crude polysaccharide test results were expressed as milligrams of glucose equivalents per gram (mg DE/g).

#### 2.4.4. Terpenoid Content

Terpenoid content was measured using the vanillin-acetic acid method, following the protocol described by Brenton et al. [17,18]. An appropriate volume of the sample extract was evaporated to dryness in a water bath at 85–100 °C. Vanillin-acetic acid solution and H_2_SO_4_ were added sequentially. Heated in a 60 °C-water bath for 30 min, then transferred to an ice bath with an appropriate amount of glacial acetic acid for 20 min. Using ursolic acid standards in the concentration range of 0–150 mg/L (R^2^ = 0.997) as a reference, with measurements at 548 nm. The detection results for terpenoid compounds were expressed as milligrams of ursolic acid equivalents per gram (mg UAE/g).

#### 2.4.5. Proanthocyanidin Content

Proanthocyanidins in avocado were detected using the hydrochloric acid-n-butanol method, following the protocol of Chen et al. [19]. Sequentially add an appropriate amount of extract, ferrous ammonium sulfate, and hydrochloric acid-n-butanol (95:5 *v*/*v*) solution. Shake well, seal, and maintain at 100 °C in a forced-air drying oven (DHG-9000, Shanghai, China) for 40 min. Cooled in an ice-water bath for 15 min, then measured at 546 nm. Using procyanidins (PC) in a concentration range of 0–1 mg/mL as the standard (R^2^ = 0.998). Procyanidins detection results are expressed as milligrams of procyanidins equivalent per gram (mg PC/g).

#### 2.4.6. Tannic Acid Content

Tannic acid in avocado was determined using the Folin-Denis method, following the protocol of Mostafa et al. [20,21]. The extracted sample solution was stepwise mixed with Folin-Denis reagent and Na_2_CO_3_ for 2 h. Using gallic acid standards in the range of 0–300 mg/L (R^2^ = 0.998) as a reference, with measurements at 765 nm. Tannic acid content was expressed as milligrams of gallic acid equivalent per gram (mg GAE/g).

#### 2.4.7. Determination of Catechin and Quercetin Content

The determination methods for catechin and quercetin content were adapted from Georgia et al., with slight modifications to the mobile phase and specific experimental procedures [22,23]. The dried sample was dissolved in 80% (*v*/*v*) methanol and refluxed at 90 °C for 4 h. Degreased, centrifuged, and filtered through a 0.45 µm filter. Determined by a Shimadzu 3000 HPLC system (Japan). A WondaSil-C18 column (4.6 mm × 250 mm, 5 µm, Japan) was employed at a column temperature of 35 °C, flow rate of 0.8 mL/min, and injection volume of 10 µL. The mobile phase consisted of ultrapure water (phase A), methanol (phase B), and 0.1% (*v*/*v*) formic acid (phase C), and the total run time per sample was 10 min. The optimal testing conditions for catechins were finalized as follows: initial gradient conditions of 64:20:16 (A:B:C), in 5 min with Phase A decreasing to 52%, Phase B increasing to 35%, and Phase C decreasing to 13% with a 5 min hold, measurements at 280 nm. The optimal testing conditions for quercetin were initial gradient conditions of 58:40:12 (A:B:C), with phase A decreasing to 20%, phase B increasing to 75%, and phase C decreasing to 5% for 10 min, measurements at 360 nm.

### 2.5. Antioxidant Assay

#### 2.5.1. DPPH Scavenging Activity (DPPH)

The DPPH scavenging rate method followed Daryna et al. [24,25]. After reacting the sample with an equal volume of 0.2 mmol/L DPPH ethanol solution in the dark for 30 min, with measurements at 517 nm. The extract solvent served as the blank control, while L-ascorbic acid at the same concentration acted as the positive control. Results are expressed as DPPH scavenging rate using the following formula:

(1) $$DPPH\;Scavenging(\%)=(1-\frac{\left(A_{s}-A_{0}\right)}{A_{b}})\times 100,$$

where A_b_ is absorbance after reacting the solvent with DPPH ethanol solution;

A_0_ is absorbance after reacting the sample extract with the solvent;
A_s_ is absorbance after reacting the sample extract with DPPH ethanol solution.

#### 2.5.2. ABTS^•+^ Scavenging Capacity (ABTS)

The ABTS^•+^ scavenging rate of avocado was determined using the method referenced from Kim et al. [26]. The sample reacted with an equal volume of ABTS^•+^ working solution (absorbance 0.700 ± 0.002) in the dark for 6 min, measured at 734 nm. Using the extract solvent as the blank control, the meanings of A_b_, A_0_, and A_S_ were the same as in DPPH. Results are expressed as ABTS^•+^ radical scavenging rate using the following formula:

(2) $$ABTS\;Scavenging\left(\%\right)=(1-\frac{\left(A_{s}-A_{0}\right)}{A_{b}})\times 100.$$

#### 2.5.3. Hydroxyl Radical Scavenging Capacity (HFR)

Hydroxyl radical scavenging rate was determined using the salicylic acid method, following the protocol of Yang et al. [27]. The addition sequence was: ferrous sulfate, salicylic acid, sample extract, deionized water, and hydrogen peroxide solution, ensuring a total reaction volume of 10 mL. The mixture was thoroughly mixed and reacted at 37 °C for 30 min. Using deionized water as the blank control, the values (A_0_, A_1_, A_2_) of each sample group were measured at 510 nm. Results were expressed as hydroxyl radical scavenging rate and calculated using the following formula:

(3) $$Hydroxyl\;Radical\;Scavenging\left(\%\right)=(1-\frac{A_{1}-A_{2}}{A_{0}})\times 100,$$

where A_1_ is absorbance after reacting the sample extract, deionized water, and hydrogen peroxide.

A_0_ is absorbance after reacting with deionized water and hydrogen peroxide.
A_2_ is absorbance after reacting the sample extract and deionized water.

#### 2.5.4. Superoxide Anion Scavenging Activity (SAFR)

The Pyrogallol method was employed to assess superoxide anion scavenging capacity, following the protocol described by Wang et al. [28]. The Tris-HCl solution (pH = 8.2), sample extract, and catechol solution allowed the reaction to proceed precisely for 4 min. Terminated the reaction by adding drops of HCl. Measured at 320 nm using deionized water as the blank control. Results were expressed as superoxide anion scavenging capacity and calculated using the following formula:

(4) $$Superoxide\;Anion\;Scavenging\left(\%\right)=(\frac{A_{control}-A_{sample}}{A_{control}})\times 100.$$

#### 2.5.5. Oxygen Radical Absorbance Capacity (ORAC)

The ORAC assay method was adapted from Acosta-Quiroga et al., with modifications to the fluorescence detection interval [29]. The sample and 0.1 µM sodium fluorescein were incubated at 37 °C for 15 min. Immediately added 0.2 M AAPH solution, began the reaction, and measured fluorescence intensity in a multi-mode microplate reader. The settings were: temperature of 37 °C, the excitation wavelength was 485 nm, the emission wavelength was 538 nm, and the measurement interval was set to 2.5 min; the total reaction time was 100 min. Using Trolox at concentrations ranging from 0 to 0.1 mmol/L as a reference, with the area under the fluorescence decay curve (AUC) at different concentrations as the *y*-axis (R^2^ = 0.997). The oxygen radical scavenging capacity of the samples was expressed in Trolox equivalents (µmol TE/g).

#### 2.5.6. Iron Reducing Antioxidant Power (FRAP)

The FRAP iron ion reduction capacity assay method followed Xiang et al. [30,31]. The sample was incubated with FRAP reagent (10 mM TPTZ:10 mM FeCl_3_:FRAP buffer = 1:1:10) in the dark at 37 °C for 30 min. Using FeSO_4_ solutions at various concentrations (0.1–10 mg/mL), with measurements at 593 nm. Sample results were expressed as FeSO_4_ equivalents per milliliter (mg FeSO_4_/mL).

### 2.6. Molecular Docking Simulation of Antioxidant Mechanisms

This study aimed to predict the binding patterns and strengths of polyphenolic compounds with biomolecules through molecular docking models, and to predict their antioxidant-related biological functions at the molecular level. At the outset of the work, protein receptors associated with antioxidant activity were first retrieved from the Protein Data Bank (PDB), while initial three-dimensional structures of ligand molecules were downloaded from the PubChem database. Using PyMOL (version 3.1.0), water molecules, existing ligands, and irrelevant ions were removed from the protein targets. Hydrogen atoms were added to the protein receptors, and AMBER charge positions were assigned. Subsequently, CB-Dock2 was employed for molecular docking, selecting the conformation with the lowest binding energy as a potential binding model for further analysis. PyMOL was used to perform non-covalent interaction analysis on the docked protein-ligand complexes, including hydrogen bonds, hydrophobic interactions, and π-π stacking. Finally, a three-dimensional visualization diagram of the interactions was generated. Finally, protein-ligand interactions were analyzed using Ligplot (version 2.3.1) and visualized as 2D plane diagrams.

### 2.7. Optimization of UAE Process Parameters Using Response Surface Methodology (RSM)

We focused on exploring the optimal combination of ultrasonication parameters with extraction solvents and other process variables to extract bioactive components from avocado seeds for applications such as natural antioxidant extracts. We systematically investigated the synergistic interactions among four factors: ethanol concentration (A), ultrasonic duration (B), ultrasonic power (C), and temperature (D), aiming to determine optimal process parameters for bioactive extraction from avocado seeds. A central composite design was implemented using Design-Expert (version 13.0), with three levels set for each process parameter. The model comprised 30 experimental combinations, each replicated at least three times. Post-analysis ANOVA testing was conducted, with *p* < 0.05 considered statistically significant.

### 2.8. Statistical Analysis

Data analysis was conducted using at least three replicates per sample. Data are presented as mean ± standard deviation (M ± SD). Data analysis was performed using SPSS Statistical Software (Version 27.0). One-way analysis of variance (ANOVA) was conducted on the data results, revealing significant differences between groups (*p* < 0.05). Tukey’s HSD post hoc multiple comparison test was performed pairwise between groups. Homogeneity of variance was assessed using Duncan’s test.

## 3. Results and Discussion

### 3.1. Nutritional Component Analysis

This study evaluated the antioxidant capacity of Hass avocados (produced in Baoshan City, Yunnan Province, China) by analyzing their compositional components based on their nutritional profile. The results were presented in Table 1.

Research analysis of basic nutrients reveals that the avocado seeds exhibit an excellent nutritional composition similar to that of the fruit flesh. The protein content in seeds (2.7 g/100 g) significantly outperforms that of apples (0.3 g/100 g), bananas (1.1 g/100 g), and mulberries (1.4 g/100 g). Research indicates seeds contain more soluble fiber than pulp; consuming seeds can naturally prevent constipation and effectively guard against gastric ulcers, making seeds a priority for research and development [32]. Conversely, the peel has an excessively high fiber content, making it difficult to digest, coarse in texture, and nutritionally limited. However, this study detected an oil content of approximately 3.36% in the seeds, which was significantly lower than the result of 11.4% [33]. Notably, temperature and light exposure shape distinct dry matter content in the fruit. Lower activity of key enzymes catalyzing lipid synthesis within the seed hindered efficient conversion and accumulation of carbohydrates like starch and sugars into lipids. Test results also showed the seed’s total sugar content was approximately 8.11 g/100 g, significantly higher than in the flesh and peel. Even under high-temperature conditions, prolonged water bath treatment struggles to break the glycosidic bonds in kernel starch, making it difficult to hydrolyze starch granules into smaller sugar molecules. This may also be related to the high content of resistant starch (RS) [34]. A higher amylose content tends to aggregate into stable, dense crystalline structures, which is a decisive factor in forming high RS content. In nutrition science, RS specifically refers to starch with prebiotic properties, offering multiple benefits such as regulating blood sugar, enhancing satiety, and improving gut health. Whole grains like legumes, oats, and brown rice share similar nutritional structures with fruit seeds, leading to the recognition of seeds as possessing comparable nutritional value to whole grains.

### 3.2. Analysis of Bioactive Components

The determination results of the bioactive components in various parts of avocados were shown in Table 2.

Research findings revealed significant differences in bioactive compounds between the flesh, seed, and peel. The flesh contains approximately 1717.37 mg/100 g of terpenoids, roughly seven times higher than that in the seed and peel. The variation may be attributed to differences in oil content, as the pulp contains significantly higher lipid levels than the peel. Lipophilic terpenoids tend to accumulate in high-oil environments due to their solubility characteristics. Given that both lipid and terpenoid biosynthesis share acetyl-CoA as a common primary metabolic intermediate, a lipid-rich cellular environment provides abundant acetyl-CoA precursors and metabolic reservoirs for terpenoid synthesis via the mevalonate pathway. These terpenoids and their derivatives constitute the primary contributors to fruit aroma and flavor complexity and may also impart endogenous antioxidant properties to the pulp. Existing research also confirms that terpenoids possess bioactive properties such as anti-inflammatory and antioxidant effects. Dietary intake of terpenoids can reduce the risk of chronic diseases and cancer [35].

Notably, the polysaccharide content in the flesh also significantly exceeds that in the seed and peel, reaching approximately 0.47 g/100 g—about 15 times higher than that in the seed. This substantial disparity is likely attributable to evolutionary pressures driven by biological function. The flesh, serving as an energy reserve, actively utilizes the glucan pathway to continuously convert dietary fibers, such as mannans and galactans. It secretes pectin esterases to break down pectin within cell walls, thereby increasing soluble polysaccharide levels and contributing to tissue softening during ripening. In contrast, the peel is naturally selected as an outer protective shell, enriched with structural polysaccharides like cellulose, most of which are insoluble and provide mechanical strength. The seed, serving as a reproductive reserve, directs its energy storage toward fatty acid synthesis rather than polysaccharide synthesis, using structural polysaccharides to protect the embryo.

This study revealed that avocado seeds and peels contain exceptionally high levels of polyphenolic compounds, approximately 5–10 times higher than the flesh, making them an excellent natural source of phenolic substances. The low phenolic compound content in flesh may stem from its metabolomic mechanisms. During flesh ripening, the bHLH transcription factor governing flavonoid synthesis pathways is suppressed, while gene networks controlling sugar metabolism and other processes are activated. Additionally, polyphenol oxidase (PPO) in the flesh degrades and converts phenolic compounds during softening to reduce astringency. Phenolic compounds not only combat pests and diseases but also resist pathogen invasion through their high antioxidant capacity, thereby maintaining dormancy and vitality. Therefore, during fruit development, phenylalanine ammonia-lyase (PAL) activity increases in seeds and peels, activating the phenylpropanoid synthesis pathway (precursor pathway for all phenolic compounds). Condensed tannins and related polyphenols confer strong bitterness and mild toxicity, serving as chemical deterrents against herbivory and predation.

Notably, the quercetin content in the avocado seed was comparable to that of yellow onions—the “king of quercetin”—while no quercetin was detected in the flesh. The seeds and peels also constituted a rich reservoir of catechins, with a content of 4.12–4.45 mg/g—six times that of the flesh—comparable to commercially available high-quality tea leaves, which was similar to that reported by Chen et al. (4.87 mg/g) [36]. The procyanidin content in avocado seeds was not only 13 times higher than that of flesh but also significantly exceeded that of berry fruits like blueberries (200 mg/100 g) and black grapes (117 mg/100 g). Moreover, polyphenolic compounds are highly valuable functional ingredients. Phenolic substances in fruits reduce CVD risk by controlling and inhibiting cellular inflammation and oxidative stress, enhancing blood flow, and suppressing platelet aggregation. Dietary supplementation with natural phenolics thus helps maintain vascular health and alleviate liver fibrosis and inflammation [37]. Therefore, this study will subsequently focus on investigating the impact of polyphenolic substances on antioxidant activity and their potential mechanisms.

### 3.3. Evaluation of Antioxidant Activity

#### 3.3.1. Correlation Analysis Between Bioactive Compound Content and Antioxidant Capacity

**Figure 1:**
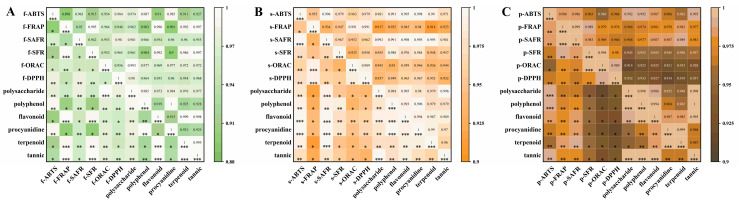
A Pearson correlation heatmap showing the relationships between polysaccharide, polyphenol, flavonoid, procyanidin, terpenoid, and tannin content and antioxidant indices (ABTS, FRAP, SAFR, SFR, ORAC, DPPH). (**A**–**C**), from left to right, represented the flesh, seed, and peel, respectively. All correlations were statistically significant (*p* < 0.05), with the intensity of the color indicating the strength of the correlation coefficient r.” to “All correlations were statistically significant (*p* < 0.05), * (*p* < 0.05), ** (*p* < 0.01), *** (*p* < 0.001), with the intensity of the color indicating the strength of the correlation coefficient r.

We employed multivariate analysis to investigate the Pearson correlation between content and activity to determine the correlation between bioactive compound content in avocado seeds and antioxidant efficacy. The correlation results between active compound content in various avocado parts and antioxidant capacity are shown in Figure 1.

All bioactive compounds measured in this study exhibited strong positive correlations with antioxidant activity indicators, all reaching statistical significance at *p* < 0.05. The Pearson correlation coefficient between fruit seed and peel exceeded 0.9. As shown in Figure 1, the content of polyphenols and flavonoid compounds exhibited strong positive correlations with all antioxidant indicators. The content of these substances largely determined the strength of antioxidant activity, consistent with the findings of Jessica et al. [38]. Although both active compound content and antioxidant activity showed positive correlations, the strength and the significance level of these correlations varied significantly. Differences in polysaccharides and polyphenolic compounds were more pronounced across different avocado parts, while flavonoids exhibited the highest *r* values and the most significant contribution. Therefore, it was inferred that changes in their content can accurately predict variations in antioxidant activity and underlying mechanisms.

Experimental results revealed that avocado by-products exhibit significant antioxidant activity against DPPH, ABTS^•+^, and FRAP, with activity levels approximately 3–4 times higher than those of the flesh. However, they did not demonstrate optimal antioxidant activity in radical scavenging experiments involving HO^•^ and O_2_^•−^. While the flesh did not exhibit notable antioxidant activity in DPPH and ABTS^•+^ assays, it revealed higher efficacy in water-soluble antioxidant tests. For instance, in hydroxyl radical scavenging and superoxide anion scavenging assays, the flesh showed more pronounced antioxidant activity than the seeds and peels at lower concentrations. Analysis suggests this may result from the flesh’s rich content of water-soluble antioxidants such as vitamin E. Water extraction better preserved their bioactivity, and the pH 7.4 conditions more accurately mimic the human physiological environment, enabling it to exhibit strong antioxidant activity. The mechanism in radical scavenging experiments likely involved the flesh’s abundant antioxidants—such as vitamin E—competitively binding to HO^•^, ROO^•^ radicals, thereby protecting the body against free radical attacks.

Avocado by-products revealed exceptionally superior antioxidant activity in experiments that evaluate both water-soluble and fat-soluble antioxidants such as DPPH, ABTS^•+^, and ORAC assays. The antioxidant activity of the avocado seed was slightly superior to that of the peel. High DPPH radical scavenging rates indicate the presence of potent antioxidants capable of supplying hydrogen atoms within the sample. Such reactions were predominantly associated with hydrogen-oxygen bonds and their bond energies, particularly prevalent in polyphenolic compounds. Conversely, strong ABTS^•+^ scavenging likely stemmed from compounds possessing exceptional electron-donating capabilities. It was speculated that these active substances may feature phenolic hydroxyl groups or enol structures, which enabled direct electron reduction in radicals or metal ions. The weaker performance of avocado seeds in water-soluble antioxidant assays such as hydroxyl radical scavenging suggested this stems from the extremely rapid reaction rate of HO^•^ (rate constant approximately 10^9^ M^−1^s^−1^). The reaction rate of antioxidants in avocado seeds was significantly slower than that of salicylic acid with HO^•^, resulting in poor performance of the seeds in competitive experiments. Test results indicated that the seeds exhibit weak superoxide anion scavenging capacity. However, current FRAP experiments demonstrated that the seeds possess strong metal ion reduction capabilities, theoretically enabling them to inhibit Fenton reactions and HO^•^ generation at the source. Theoretically, they also exhibited strong O_2_^•−^ scavenging capacity. The poor analytical results may stem from the alkaline conditions used in superoxide anion scavenging experiments. Polyphenolic and flavonoid compounds typically exhibited peak activity in weakly acidic environments. Under alkaline conditions, higher OH^−^ concentrations caused phenolic hydroxyl groups to lose protons (H^+^), leading to structural disruption and functional inactivation, thereby explaining the experimental discrepancies.

The above experiments revealed that avocado by-products were highly effective direct radical scavengers, particularly excelling in hydrogen atom transfer (HAT) and electron transfer (ET) capabilities. They represented a natural source of antioxidants with specific properties. Furthermore, the superior antioxidant capacity of the seed extract in ORAC provided a basis for subsequent applications in neutral pH environments, such as physiological conditions. However, it remained elucidated which specific compounds contributed most significantly to the overall antioxidant activity and which substance groups were primarily responsible for the potent antioxidant capacity mediated by HAT and ET mechanisms. Subsequent studies will focus on the avocado seed, which exhibits optimal antioxidant activity, with particular emphasis on its remarkably pronounced DPPH radical scavenging capacity.

#### 3.3.2. Preliminary Comparison of In Vitro Antioxidant Activity in Extracts of Active Compounds from Avocado Seeds

Subsequent experiments will therefore validate the antioxidant potency of different bioactive compounds within the seeds. This aims to identify the compounds responsible for the potent antioxidant effects in avocado seeds and assess how variations in their content influence antioxidant activity.

Plant extracts served as natural antioxidant resources, yet the antioxidant mechanisms and efficacy of different bioactive compounds within plants varied significantly. Comparing the antioxidant properties of these structurally diverse and mechanistically distinct macromolecular groups is crucial for the preliminary screening of highly effective natural antioxidants. Therefore, this experiment employed an in vitro chemical model to evaluate the antioxidant activity of extracts containing polysaccharides, polyphenols, flavonoids, procyanidins, terpenoids, and tannic acid from avocado seeds. This approach aims to assess the antioxidant potency of extracts enriched with different bioactive compounds and provided a foundation for subsequently identifying the most bioactive substances within avocado seeds.

**Figure 2:**
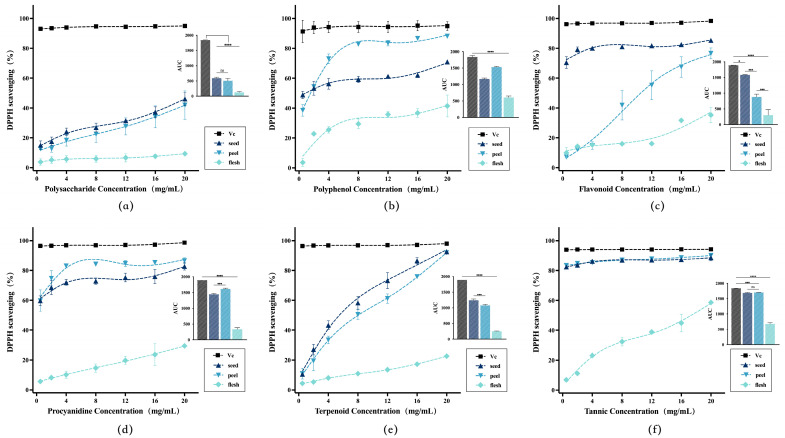
The concentration-response curves of DPPH radical scavenging activity for extracts of different bioactive compounds from avocado. Bar charts represented AUC values for different fractions, labeled sequentially as (**a**–**f**): polysaccharides, polyphenols, flavonoids, procyanidins, terpenoids, and tannic acid. * (*p* < 0.05), *** (*p* < 0.001), **** (*p* < 0.0001).

The line graph in Figure 2 revealed a clear visual representation of the antioxidant potency of different extracts, and the area under the curve (AUC) bar chart enabled a direct quantitative comparison of their overall efficacy. In the AUC bar charts for various extracts, avocado by-products consistently demonstrated significantly stronger antioxidant activity than that of the fruit pulp. Notably, even in extracts derived from procyanidins and tannins, avocado by-products exhibited total antioxidant effects approaching those of vitamin C. The results indicated that the antioxidant activity of the six extracts from avocado by-products follow this order of potency: procyanidins (Figure 2d), tannic acid (Figure 2f) > flavonoids (Figure 2c) > polyphenols (Figure 2b) > terpenoids (Figure 2e) > polysaccharides (Figure 2a). This suggested that compounds rich in phenolic hydroxyl groups can exhibit particularly strong in vitro free radical scavenging capacity, consistent with previous studies.

In this study, the flavonoid extract exhibited higher activity than the broad-spectrum polyphenol extract. As a key subclass of polyphenols, flavonoids often featured structures containing ortho-dihydroxyl groups and other cross-conjugated systems. It was hypothesized that the flavonoid extract may be rich in conjugated flavonoid monomers such as procyanidins, quercetin, and catechin, thereby enhancing its overall activity.

The antioxidant mechanism of terpenoids may not be based on electron and hydrogen atom transfer. Terpenoids activated intracellular antioxidant signaling pathways by regulating gene expression of HO-1, NQO1, and others, but their lack of in vitro antioxidant activity in this experiment [39]. Polysaccharides exhibited relatively weak direct radical scavenging capacity; typically exerting their effects by activating macrophages and enhancing superoxide dismutase (SOD) activity in vivo [40]. Procyanidin extracts demonstrated significantly higher antioxidant activity than other categories in additional antioxidant assay models. This suggested that procyanidins likely contribute most substantially to antioxidant activity in avocado seeds. However, the efficacy of broad-spectrum active substance extracts may result from the average effect of both high- and low-activity components acting together. In contrast, procyanidin extracts exhibited greater uniformity and specificity. The total number of active phenolic hydroxyl groups in procyanidin extracts exceeded that in conventional broad-spectrum extracts. The large quantity of phenolic hydroxyl groups likely generated synergistic antioxidant effects through hydrogen bonding interactions and conjugated effects between aromatic rings.

Although the research results revealed significant variability, differences in antioxidant activity at the same extraction concentration were likely due to variations in the content of active ingredients per unit mass, rather than differences in the active components themselves. To eliminate concentration interference and infer the intrinsic strength of antioxidant efficacy, subsequent experiments will further analyze the relationship between antioxidant activity and content.

#### 3.3.3. Analysis of Antioxidant Potency of Active Compounds in Fruit Seeds

**Figure 3:**
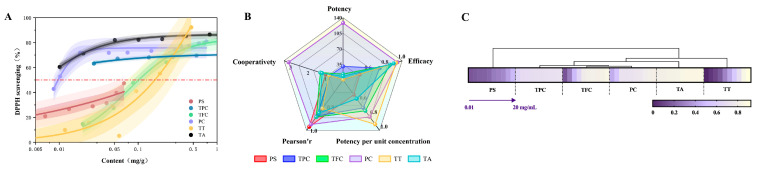
(**A**): The dose–response curves for extracts of different bioactive compounds from avocado seeds. (**B**): A comprehensive antioxidant radar chart. (**C**): The heatmap of DPPH Radical Scavenging Activity. PS: polysaccharides, TPC: total polyphenols, TFC: total flavonoids, PC: proanthocyanidins, TT: terpenoids, and TA: tannic acid.

This study further employed the Hill model to investigate the underlying patterns. The EC_50_ and maximum effective concentrations of active compounds in the seeds were estimated, and a cluster analysis was conducted on the detected active compounds to explore the relationship between the content of multiple active compounds and antioxidant indicators. The results are presented in Figure 3.

To quantify the antioxidant potency of different substances, we performed Hill model fitting with active substance content as the independent variable and antioxidant activity as the dependent variable, as shown in Figure 3A. The fitting results revealed that procyanidins and tannic acid extracts exhibited the lowest EC_50_ values, suggesting that even at equivalent active substance contents, procyanidins also demonstrate superior antioxidant effects compared to other substances. The Hill coefficient *n* of the procyanidin fitting curve was approximately 3.1, significantly greater than 1. This revealed a potential positive synergistic effect between or within procyanidin molecules: the reaction of one active site with free radicals may enhance the reactivity of other sites within the molecule, leading to an exponential increase in binding efficiency.

As shown in Figure 3B, the procyanidin extract exhibited the largest and most balanced radar chart area, with significant extension across all indicators. This revealed that procyanidins possessed the strongest comprehensive antioxidant capacity among substances in avocado seeds, acting as broad-spectrum, highly efficient antioxidants. In contrast, the polysaccharide extract exhibited the smallest radar chart area with a compact shape, suggesting weaker direct antioxidant activity in vitro. This strongly revealed that polysaccharides likely possess selective indirect antioxidant activity.

As shown in Figure 3C, the correlation heatmap visually displayed the relative levels of active compounds extracted from avocado seeds under the same indicator. The rows corresponding to procyanidins and tannic acid extracts exhibited the lightest colors, indicating their highest antioxidant values. In contrast, the rows for polysaccharide extracts and terpenoid extracts were predominantly purple, signifying their relatively weaker in vitro antioxidant capacity. Cluster analysis grouped procyanidins and flavonoid extracts into one cluster, which showed closer proximity to polyphenol and tannic acid extracts. This revealed their shared chemical and functional characteristics as phenolic compounds. In contrast, polysaccharide extracts form a distinct cluster, showing the greatest divergence from other categories. This aligned with the unique, variable structure of polysaccharides and their indirect antioxidant mechanisms.

Procyanidins’ exceptionally high potency and strong synergistic effects provide a solid theoretical basis for their development as natural antioxidants. However, in vitro antioxidant models cannot fully replicate the complex physiological environment within the body. The ability of these extracts to activate antioxidant pathways in vivo may be even more crucial. Therefore, the next step will involve molecular docking simulations to investigate their role in activating antioxidant pathways in the human body.

### 3.4. Molecular Docking Simulation of Antioxidant Mechanisms

Flavonoids constituted the most significant contributors to antioxidant activity within avocado seeds. Therefore, this study selected and compared the binding patterns of procyanidins, quercetin, and catechin—flavonoids abundant in seeds—with antioxidant pathway proteins in the human body to elucidate the mechanism underlying procyanidins’ potent activity. This study focused on two protein targets associated with human antioxidant pathways. The first target protein (PDB ID: 6W7O) is located in the cytoplasmic domain of NADPH oxidase 2 (NOX2). Where NOX2 is a key enzyme generating reactive oxygen species (ROS), it catalyzes NADPH into damaging ROS like superoxide anion and hydrogen peroxide, while also serving as a crucial activator for pro-inflammatory signaling pathways such as NF-κB. We revealed that procyanidins bind to NOX2’s protein subunits, may inhibiting its catalytic activity and reducing ROS production at its source. Furthermore, bioactive compounds in avocado seeds serve as potent electron donors, may converting NOX2’s downstream ROS products into harmless water or oxygen, thereby alleviating oxidative stress.

**Figure 4:**
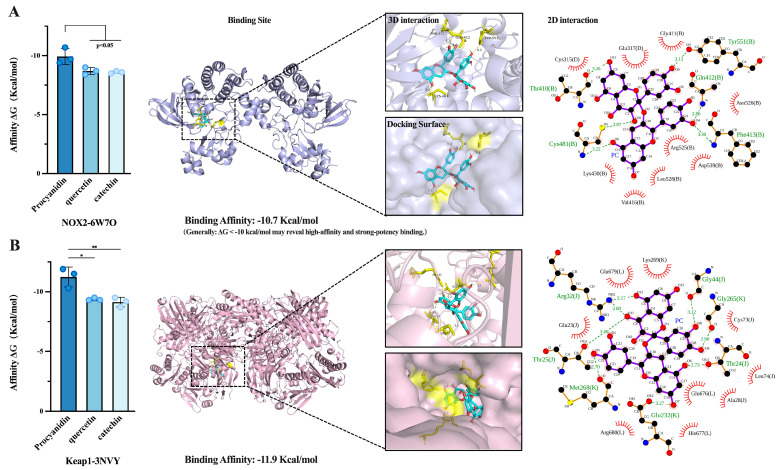
(**A**): Schematic diagram of the interaction between 6W7O-procyanidin (blue stick model). (**B**): Schematic diagram of the interaction between 3NVY and procyanidin. Bar charts: The binding energies of different ligands with proteins, * (*p* < 0.05), ** (*p* < 0.01). Enlarged view of the binding site: Key amino acid residues (yellow sticks), hydrogen bonds (yellow dashed lines). 2D interaction diagram: Ligand (purple compound), hydrogen bonds (green dashed lines), hydrophobic interactions (red sunflower-like structures).

The second protein target is the target protein located within the Kelch domain of the Keap1 protein (PDB ID: 3NVY). Keap1 serves as a key negative regulator in the Keap1-Nrf2 pathway. Under low oxidative stress conditions, Keap1 binds to cytoplasmic Nrf2 and mediates its ubiquitination and degradation via the proteasome. This maintains Nrf2 at low levels, preventing excessive activation of cellular antioxidant responses. Research reveals that procyanidins may inhibit Nrf2 degradation by binding to Keap1, thereby initiating the expression of the endogenous antioxidant system. This study aims to theoretically elucidate the binding site and mode of procyanidins with the Keap1 protein through molecular docking, thereby predicting the potential mechanism for activating the Nrf2 pathway. Molecular docking simulation results and binding energies are shown in Figure 4.

This study compared the antioxidant capacities and potential mechanisms of action by analyzing the binding energy, binding patterns, and interaction forces between procyanidins, quercetin, catechin, and the protein targets 6W7O and 3NVY. Based on binding energy analysis, procyanidins exhibited superior binding affinity and stability toward these two antioxidant targets compared to quercetin and catechin. In the field of medicinal chemistry, a molecular docking binding energy ΔG < −10 kcal/mol typically indicates strong protein binding, high affinity, and prolonged binding residence time with the compound. It suggests potential sub-micromolar biological activity. The binding energies between procyanidins and their protein targets are all below −10 kcal/mol. From a physiological perspective, it reveals that procyanidins are likely effective inhibitors/activators of these protein targets.

Observation of procyanidin’s optimal binding mode revealed that the complex-protein system in the 6W7O conformation was thermodynamically highly stable. The binding pattern between procyanidin and its protein receptor revealed exceptional specificity. The procyanidin molecule deeply embedded within the hydrophobic channel formed by α-helices in NOX2. The binding pocket exhibited a cavernous structure, demonstrating high spatial complementarity with the overall three-dimensional conformation of procyanidin, indicating its high affinity. As shown in Figure 4A, the procyanidin molecule is completely surrounded by surrounding amino acid residues. It primarily interacts with residues such as Cys481, Tyr551, and Gln412 in the protein pocket via hydrogen bonds, forming a relatively stable hydrogen bond network. Strong hydrophobic interactions also exist between Leu528 residues and procyanidin, creating a stable hydrophobic core. This deeply embedded binding mode significantly restricts the translational and rotational freedom of procyanidins within the pocket. It may competitively inhibit protein binding to the NOX2 enzyme through steric hindrance effects, theoretically elucidating the structural basis for its antioxidant activity.

According to molecular docking results, the binding energy between procyanidin and the 3NVY protein target in the Keap1 pathway is as low as −11.7 kcal/mol, which may reveal extremely strong binding affinity between them. As shown in the overall structure (Figure 4B), procyanidin is deeply embedded into a hydrophobic cavity formed by an α-helix and β-sheet, a structure typically serving as the key binding site for high-affinity ligands. This cavity-like binding site tightly encloses the procyanidin molecule. Procyanidin may directly bond to cysteine residues on the Keap1 protein, triggering a Michael addition reaction. This could induce a conformational change in Keap1, thereby inhibiting its ability to degrade Nrf2. Consequently, Nrf2 may bind to the antioxidant response element (ARE) within the cell nucleus, initiating the expression of a series of antioxidant genes. As shown in Figure 4B, Lys73 and Arg32 within the Keap1 receptor exhibit exceptionally tight binding to procyanidins. Most of these amino acids carry positive charges under physiological conditions, suggesting procyanidins may serve as excellent electron donors. The tricyclic aromatic structures of procyanidins form strong π-cation interactions with amino acid residues in the proteins, significantly enhancing binding stability. Specifically, the guanidino group of residues Arg32 forms a potent interaction with the π electron cloud of procyanidin’s A ring, with a center-of-mass distance of approximately 2Å, constituting a strong binding. Furthermore, the B ring of procyanidin forms T-shaped π-π stacking with Lys73. These pronounced π-system interactions, combined with extensive hydrophobic interactions and hydrogen bonds, collectively confer a highly specific binding mode to procyanidin. Theoretical analysis suggests this enables potential modification of the Keap1 protein, indirectly activating the Nrf2 pathway to initiate an endogenous, comprehensive antioxidant stress defense program within cells.

It must be emphasized that molecular docking simulations are limited to theoretical mechanism studies and serve just predictive results to assist in inferring the antioxidant mechanism of procyanidins. Subsequent studies will establish animal models of oxidative stress and conduct relevant cellular experiments to evaluate comprehensively the antioxidant biological efficacy of procyanidins.

### 3.5. RSM Method for Optimizing the Extraction Process

This study employed ultrasonic-assisted extraction (UAE) technology using ethanol-water solvents. Response surface methodology was applied to optimize the extraction process, investigating the effects of four factors—ethanol concentration (A), ultrasonic duration (B), ultrasonic power (C), and temperature (D)—on antioxidant activity. It determined optimal extraction conditions to yield extracts with maximum antioxidant potency.

**Figure 5:**
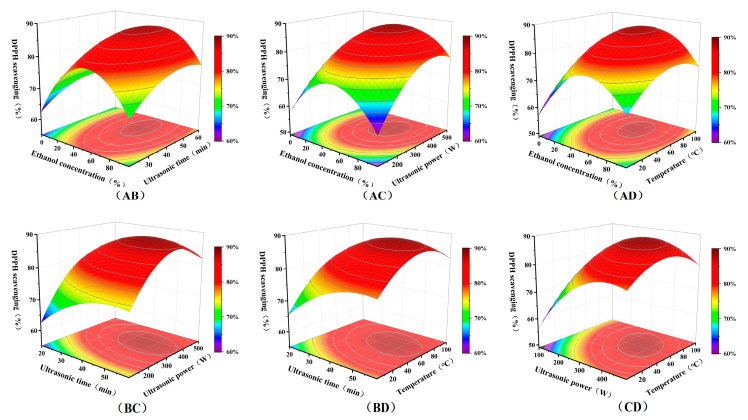
The interaction between ethanol concentration (A), ultrasonication time (B), ultrasonic power (C), and temperature (D).

We obtained a quadratic regression model with DPPH radical scavenging rate as the target response. Table 3 and Table 4 present the relevant experimental parameters of the model. According to the analysis of variance (ANOVA) in Table 4, the model exhibited a highly significant *p*-value, while the misfit term was insignificant. This indicated the model fits the experimental data well with minimal error. Both R^2^ and R^2^_adj_ values exceeded 0.9, signifying the model can explain over 90% of the response variation and possesses accurate predictive capability. The linear and quadratic effects of each variable on the response were highly significant, indicating an interactive effect among these four factors on the extraction process. Among these, ethanol concentration (A), ultrasonic power (C), and temperature (D) exerted the most significant effects on DPPH radical scavenging rate. This aligned with the principle of “like dissolves like” and indicated that the active substances responsible for antioxidant efficacy were relatively sensitive to temperature. Figure 5 presents the four-factor, three-level interaction response surface plot generated using Design-Expert (version 13.0) based on the aforementioned conditions.

The response surface plot (Figure 5) visually revealed the interactions among the four process parameters and their effects on antioxidant activity. The response surfaces for the interactions between ethanol concentration and ultrasonication time, ultrasonic power, and temperature showed distinct peaks (deep red areas in the figure). At low ethanol concentrations, increasing ultrasonication time and power yields limited improvements in antioxidant activity. This may result from the solvent’s strong polarity, which reduces extraction efficiency for polyphenolic compounds. Conversely, excessively high ethanol concentrations may cause plant cell wall denaturation and protein coagulation, hindering the dissolution of large polyphenolic molecules. The analysis revealed that solvent concentration likely exerted the greatest influence on the dissolution of bioactive compounds within seeds. At moderate ethanol concentrations (40–70%), the antioxidant activity of the extract remained at a high level. It was likely because this concentration maximizes the dissolution of polyphenols and flavonoids, while the moderately polar solvent water also dissolved some water-soluble substances.

At optimal ethanol concentrations, increasing ultrasonic power and temperature significantly enhanced the antioxidant activity of the extract. Analysis suggested that appropriately elevated ultrasonic intensity generates stronger vortex and mixing effects, thereby markedly improving mass transfer rates. However, excessively high power may induce intense cavitation effects, leading to degradation of bioactive compounds. Moderately increasing temperature can enhance antioxidant activity. This effect was likely attributed to thermal assistance reducing solvent viscosity and surface tension, thereby promoting mutual permeation between the solvents and materials. Conversely, excessively high temperatures may degrade heat-sensitive bioactive compounds, resulting in a significant decline in antioxidant activity. The model predicted optimal process parameters for the antioxidant extract were 60% ethanol, 45 min, ultrasonic power of 350 W, and 55 °C. Under these conditions, the DPPH radical scavenging rate of the seed extract could reach 91.24%, closely matching the model prediction (89.2%). This was likely attributed to the high solvent solubility at this concentration, where moderate ultrasonic power and temperature enabled efficient extraction while minimizing thermal degradation of bioactive compounds, yielding the extract with the highest antioxidant activity. Furthermore, the use of ethanol-water in this study offered significant advantages over traditional solvents in terms of safety and environmental friendliness.

While the present study successfully optimized ultrasound-assisted extraction (UAE) of avocado seed antioxidants using ethanol-water mixtures, it should be noted that UAE remains primarily a laboratory-scale technique with limited scalability. To achieve industrial relevance, future research should explore more scalable, energy-efficient, and sustainable approaches. In this regard, hydrodynamic cavitation has recently emerged as a promising green technology for large-scale extraction of bioactive compounds, combining high efficiency, water-based operation, and industrial feasibility [9]. Hydrodynamic cavitation is emerging as one of the most promising and innovative technologies for extracting natural products from entire plant materials, including by-products from supply and processing chains [41]. Integrating such technologies could significantly enhance the sustainability and applicability of antioxidant recovery from avocado by-products.

## 4. Conclusions

The study successfully developed an effective and safe extraction process for avocado by-products using a multidimensional optimization approach—60% ethanol, 45 min ultrasonication, 350 W ultrasonic power, and 55 °C temperature—while elucidating the underlying antioxidant mechanisms. These mechanisms are primarily attributed to bioactive constituents such as procyanidins, which directly scavenge free radicals through hydrogen atom transfer (HAT) and electron transfer (ET) pathways. Molecular docking analyses further confirm their strong binding affinity for Keap1 and NOX2 proteins, suggesting potential activation of the Nrf2 signaling pathway and inhibition of reactive oxygen species (ROS) generation. The results demonstrate that avocado seed extract is a high-quality natural antioxidant, exhibiting both direct free radical scavenging capacity and indirect potential to upregulate cellular defense systems. This work establishes a comprehensive technical and theoretical foundation for valorizing similar agro-industrial by-products in food, pharmaceutical, and cosmetic applications. Future studies will focus on in-depth structure–activity relationship analysis of individual compounds to further elucidate their antioxidant mechanisms, as well as in vivo functional validation experiments to confirm their biological effects. Additionally, the translation of optimized extraction conditions to industrial-scale applications will be explored, with potential evaluation of emerging green technologies such as hydrodynamic cavitation to enhance process sustainability and scalability.

## Figures and Tables

**Table 1:** The Nutritional Components of Avocados (M ± SD, *n* = 3).

Name	Moisture (%)	Ash (%)	Crude Liquid (%)	Crude Fiber (%)	Total Acid (%)	Protein (%)	Total Sugar (g/100 g)
flesh	75.63 ± 0.81 a	1.64 ± 0.38 a	18.95 ± 0.22 a	2.53 ± 0.19 c	1.14 ± 0.09 a	2.28 ± 0.07 b	3.31 ± 0.26 b
seed	52.37 ± 1.21 c	1.24 ± 0.32 ab	3.36 ± 0.06 b	5.58 ± 0.90 b	1.18 ± 0.06 a	2.77 ± 0.15 a	8.11 ± 0.76 a
peel	64.45 ± 1.63 b	0.85 ± 0.02 b	2.68 ± 0.03 c	16.33 ± 0.24 a	0.73 ± 0.04 b	1.60 ± 0.14 c	3.13 ± 0.72 b

Calculations are based on fresh weight (FW). The letters (a, b, c) next to parameters indicate statistically significant differences (*p* < 0.05).

**Table 2:** Active ingredient content of avocados (M ± SD, *n* = 3).

	Total Polyphenol (mg/100 g)	Flavonoid (mg/100 g)	Polysaccharose (g/100 g)	Procyanidin (mg/100 g)	Terpenoid (mg/100 g)	Tannic (mg/100 g)	Catechin (mg/g)	Quercetin (ug/g)
flesh	240.70 ± 23.98 c	255.37 ± 22.01 c	0.47 ± 0.00 a	34.08 ± 1.08 c	1717.37 ± 157.19 a	356.63 ± 3.61 b	0.70 ± 0.03 b	-
seed	2343.61 ± 31.44 a	2645.59 ± 84.68 a	0.03 ± 0.01 c	485.76 ± 15.84 a	241.20 ± 2.41 b	1043.33 ± 24.13 a	4.45 ± 0.61 a	259.49 ± 44.35 a
peel	1055.23 ± 30.97 b	521.49 ± 14.57 b	0.16 ± 0.01 b	281.39 ± 11.23 b	182.28 ± 9.98 b	1040.51 ± 7.77 a	4.12 ± 1.77 a	178.19 ± 15.85 b

Calculations were based on fresh weight (FW), where “-” indicated not detected. Different letters (a, b, c) indicate significant differences between data points.

**Table 3:** CCD response surface design plan and experimental results.

Run	A	B	C	D	Actual	Predicted	Residual
1	0 (−1)	20 (−1)	100 (−1)	20 (−1)	0.25868	0.2789	−2.02%
2	100 (1)	20 (−1)	100 (−1)	20 (−1)	0.315794	0.3192	−0.35%
3	0 (−1)	60 (1)	100 (−1)	20 (−1)	0.42033	0.4267	−0.64%
4	100 (1)	60 (1)	100 (−1)	20 (−1)	0.501974	0.4901	1.18%
5	0 (−1)	20 (−1)	500 (1)	20 (−1)	0.47531	0.4917	−1.64%
6	100 (1)	20 (−1)	500 (1)	20 (−1)	0.625494	0.6318	−0.63%
7	0 (−1)	60 (1)	500 (1)	20 (−1)	0.5933	0.5554	3.79%
8	100 (1)	60 (1)	500 (1)	20 (−1)	0.688426	0.7184	−3.00%
9	0 (−1)	20 (−1)	100 (−1)	100 (1)	0.590798	0.5461	4.47%
10	100 (1)	20 (−1)	100 (−1)	100 (1)	0.506254	0.5373	−3.11%
11	0 (−1)	60 (1)	100 (−1)	100 (1)	0.605521	0.5924	1.31%
12	100 (1)	60 (1)	100 (−1)	100 (1)	0.637822	0.6067	3.11%
13	0 (−1)	20 (−1)	500 (1)	100 (1)	0.565849	0.5709	−0.50%
14	100 (1)	20 (−1)	500 (1)	100 (1)	0.682848	0.6618	2.11%
15	0 (−1)	60 (1)	500 (1)	100 (1)	0.551169	0.533	1.81%
16	100 (1)	60 (1)	500 (1)	100 (1)	0.673935	0.6469	2.70%
17	0 (−1)	40 (0)	300 (0)	60 (0)	0.21579	0.2379	−2.22%
18	100 (1)	40 (0)	300 (0)	60 (0)	0.392891	0.3922	0.06%
19	50 (0)	20 (−1)	300 (0)	60 (0)	0.652859	0.6338	1.91%
20	50 (0)	60 (1)	300 (0)	60 (0)	0.726293	0.7669	−4.06%
21	50 (0)	40 (0)	100 (−1)	60 (0)	0.391613	0.4007	−0.90%
22	50 (0)	40 (0)	500 (1)	60 (0)	0.641289	0.6537	−1.25%
23	50 (0)	40 (0)	300 (0)	20 (−1)	0.538643	0.5114	2.73%
24	50 (0)	40 (0)	300 (0)	100 (1)	0.658328	0.7071	−4.88%
25	50 (0)	40 (0)	300 (0)	60 (0)	0.858378	0.8707	−1.23%
26	50 (0)	40 (0)	300 (0)	60 (0)	0.884378	0.8707	1.37%
27	50 (0)	40 (0)	300 (0)	60 (0)	0.878805	0.8707	0.81%
28	50 (0)	40 (0)	300 (0)	60 (0)	0.884853	0.8707	1.41%
29	50 (0)	40 (0)	300 (0)	60 (0)	0.885875	0.8707	1.52%
30	50 (0)	40 (0)	300 (0)	60 (0)	0.881995	0.8707	1.13%

**Table 4:** Variance analysis (ANOVA) of CCD model.

Source	Sum of Squares	df	Mean Square	*F*-Value	*p*-Value
Model	0.9754	14	0.0697	53.78	<0.0001 ***
A-Ethanol concentration	0.0357	1	0.0357	27.57	<0.0001 ***
B-Ultrasonic time	0.0266	1	0.0266	20.50	0.0004 **
C-Ultrasonic power	0.0961	1	0.0961	74.16	<0.0001 ***
D-Temperature	0.0575	1	0.0575	44.35	<0.0001 ***
AB	0.0005	1	0.0005	0.4091	0.5321
AC	0.0099	1	0.0099	7.66	0.0143 *
AD	0.0024	1	0.0024	1.86	0.1923
BC	0.0071	1	0.0071	5.47	0.0336 *
BD	0.0103	1	0.0103	7.95	0.0129 *
CD	0.0354	1	0.0354	27.31	0.0001 **
A^2^	0.5292	1	0.5292	408.52	<0.0001 ***
B^2^	0.0498	1	0.0498	38.42	<0.0001 ***
C^2^	0.2023	1	0.2023	156.15	<0.0001 ***
D^2^	0.1172	1	0.1172	90.47	<0.0001 ***
Residual	0.0194	15	0.0013		
Lack of Fit	0.0147	10	0.0015	1.57	0.3231
Pure Error	0.0047	5	0.0009		
Cor Total	0.9948	29			

Y = −0.093949 + 0.005596 (A) + 0.012742 (B) + 0.001926 (C) + 0.006265 (D) + 0.000006 (AB) + 0.000003 (AC) − 0.000005 (AD) − 0.000005 (BC) − 0.000025 (BD) − 0.000005 (CD) − 0.000056 (A^2^) − 0.000107 (B^2^) − 0.000002 (C^2^) − 0.000026 (D^2^); Where R^2^ = 0.9805, R^2^_adj_ = 0.9622, *p* < 0.05 was denoted as *, *p* < 0.01 as **, and *p* < 0.001 as ***.

## Data Availability

The original contributions presented in the study are included in the article; further inquiries can be directed to the corresponding author.

## Abbreviations

The following abbreviations are used in this manuscript:

DPPH	DPPH Scavenging
ABTS	ABTS Scavenging
FRAP	FRAP Ion Reducing Antioxidant Power
HFR	Hydroxyl Radical Scavenging
SAFR	Superoxide Anion Scavenging
ORAC	Oxygen Radical Absorbance Capacity
PS	Polysaccharide
TPC	Total Phenolic Content
TFC	Total Flavonoid Content
PC	Procyanidin
TT	Terpenoid
TA	Tannic acid
